# The dielectric response of phenothiazine-based glass-formers with different molecular complexity

**DOI:** 10.1038/s41598-021-95127-y

**Published:** 2021-08-04

**Authors:** M. Rams-Baron, A. Jędrzejowska, K. Jurkiewicz, M. Matussek, M. Musiał, M. Paluch

**Affiliations:** 1 August Chełkowski Institute of Physics, University of Silesia in Katowice, 75 Pułku Piechoty 1, 41-500 Chorzów, Poland; 2 Silesian Center for Education and Interdisciplinary Research, 75 Pułku Piechoty 1a, 41-500 Chorzów, Poland; 3 PET Diagnostics Department, Maria Sklodowska-Curie Memorial Cancer Centre and Institute of Oncology, Gliwice Branch, Gliwice, Poland; 4 Institute of Chemistry, University of Silesia in Katowice, Szkolna 9, 40-006 Katowice, Poland

**Keywords:** Chemistry, Physics

## Abstract

We examined a series of structurally related glass-forming liquids in which a phenothiazine-based tricyclic core (PTZ) was modified by attaching *n*-alkyl chains of different lengths (*n* = 4, 8, 10). We systematically disentangled the impact of chemical structure modification on the intermolecular organization and molecular dynamics probed by broadband dielectric spectroscopy (BDS). X-ray diffraction (XRD) patterns evidenced that all PTZ-derivatives are not ‘ordinary’ liquids and form nanoscale clusters. The chain length has a decisive impact on properties, exerting a plasticizing effect on the dynamics. Its elongation decreases glass transition temperature with slight impact on fragility. The increase in the medium-range order was manifested as a broadening of the dielectric loss peak reflected in the lower value of stretching parameter *β*_KWW_. A disagreement with the behavior observed for non-associating liquids was found as a deviation from the anti-correlation between the value of *β*_KWW_ and the relaxation strength of the *α*-process. Besides, to explain the broadening of loss peak in PTZ with the longest (decyl) chain a slow Debye process was postulated. In contrast, the sample with the shortest alkyl chain and a less complex structure with predominant supramolecular assembly through π–π stacking exhibits no clear Debye-mode fingerprints. The possible reasons are also discussed.

## Introduction

Dielectric spectroscopy is a powerful tool for studying the dynamics of self-assembling glass-forming systems over a wide range of temperatures and frequencies and also under high pressure. Much effort is now being made to find quantitative relationships between various parameters describing the dielectric response and identify those universal for a wide group of materials^1–3^. The strongly interacting liquids with the intermolecular organization going beyond the short-range order are usually excluded from such a discussion according to the idea of ‘understanding the glass transition in its simplest form, before moving to the more general and probably more complex scenario’^4^. Indeed, in systems where we deal with any intermolecular forces (hydrogen bonds, ionic bonds, *π*–*π* stacking), the self-aggregation and nanoscale organization may result in a profound variability of their dynamics, depending on the size, architecture, and stability of the structures formed. Consequently, new phenomena and unusual dynamic behaviors compared to ordinary liquids may emerge. Thus, systematic studies allowing a deep understanding of the structure-dynamics correlations in this group of glass-forming materials are necessary. Particularly useful are investigations on structural analogs that allow in a systematic way to disentangle the effect of changes in the chemical structure on the dielectric response.

To address this problem, we have studied the relaxation dynamics of a series of phenothiazine (PTZ)-based compounds using broadband dielectric spectroscopy (BDS). PTZs are a versatile group of materials attractive for various real-life applications. They are widely used in medicine and pharmacy as leading fragments of drugs with a broad spectrum of biological activities, including antipsychotic, antihistamine, and antiseptic^5,6^. Due to remarkable electronic properties, PTZ derivatives were found to be notably useful in optoelectronics and material science^7^. The availability of the PTZ scaffold for various chemical modifications makes its derivatives a powerful platform for creating new compounds with desired properties.

**Scheme 1:**
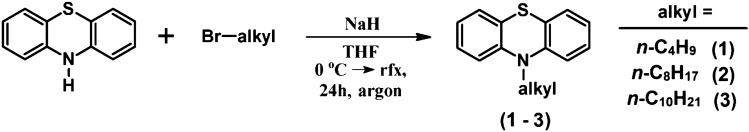
Schematic representation of the synthesis of investigated PTZs.

The goal of this study is to systematically elucidate how the structural and molecular properties, in particular differences in the intermolecular configuration, govern the dielectric response of a series of structurally related glass-forming molecules based on the PTZ backbone. We examined three PTZ derivatives with increasing alkyl chain length, i.e., butyl (assigned as PTZ-C4), octyl (PTZ-C8), and decyl (PTZ-C10). Their chemical structures are sketched in Scheme 1. The choice of these materials was dictated by differences in their affinity to supramolecular structuring revealed by X-ray diffraction (XRD). In such *π*-conjugated systems, the intermolecular *π–π* stacking interactions are believed to be the driving force behind their self-organization^8–10^. We expected that the length of the alkyl chain might have a decisive impact on the configuration of aromatic PTZ units, differentiating the observed X-ray scattering response in the low-scattering vector range covering the nanoscale structure. Indeed, the XRD results confirmed two types of supramolecular organization in the PTZs, which vary with the length of the side chain attached to the identical tricyclic core. The first one is related to the parallel *π–π* stacking of aromatic units, while the second one results from an organization of alkyl chains along their long axis. Thus, these structurally related glass-forming molecules with different self-assembling abilities are perfect materials to study structure-dynamics interplays. The question we ask is if the differences in their structure found in the XRD patterns translate into the molecular dynamics in the supercooled and glassy states probed by dielectric spectroscopy. Do we observe any peculiarities resulting from the molecular association of these systems, or is the dielectric response of PTZs similar to that registered for glass-forming liquids lacking specific interactions and a tendency to self-aggregation?

## Material and methods

### Synthetic procedures and characterization

All reagents were purchased from Sigma-Aldrich or TCI Chemicals and were used without further purification. Tetrahydrofuran was heated to reflux with sodium and benzophenone and distilled before use. Column chromatography was carried out on Merck silica gel (230–400 mesh). Thin-layer chromatography (TLC) was performed on silica gel (Merck TLC Silica Gel 60, F254). NMR spectra were taken on Bruker Avance 400 (400 MHz) spectrometer. Chemical shifts (*δ*) are reported in parts per million (ppm) relative to traces of CHCl_3_ (*δ*_H_ = 7.26 ppm, *δ*_C_ = 77.0 ppm). The key 10-alkylated PTZs were synthesized according to the well-known literature procedure with minor modifications^11^.

Phenothiazine (10 g, 50.2 mmol) was dissolved in 300 mL of anhydrous tetrahydrofuran and the solution was cooled to 0 °C using an ice bath. The reaction system was kept under an argon atmosphere. Sodium hydride (2.17 g, 90.4 mmol, 1.8 eq) was added to a solution in one portion, and the resulting mixture was stirred at room temperature for 1 h. After this time, 1-bromobutane (8.94 g, 7.1 mL, 65.3 mmol), 1-bromooctane (12.60 g, 11.3 mL, 65.3 mmol) or 1-bromodecane (14.44 g, 13.5 mL, 65.3 mmol) was added, and the resulting mixture was vigorously stirred and heated under reflux for 24 h. The progress of the reaction was monitored by thin-layer chromatography (TLC). Then, ice was added, and the mixture was extracted with ethyl acetate (3 × 150 mL). The combined organic layers were dried over MgSO_4_, filtered to remove solids, and the filtrate was evaporated under reduced pressure. The residue was purified by column chromatography.

10-butylphenotiazine (**PTZ-C4**).
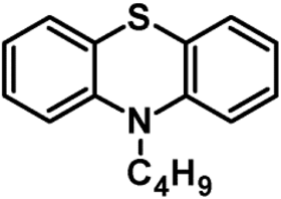


The crude product was purified by column chromatography (silica gel, hexane), to give **PTZ-C4** as a yellowish oil liquid (11.2 g, 87% yield). ^1^H NMR (400 MHz, CDCl_3_) δ: 7.21–7.08 (m, 4H), 6.96–6.82 (m, 4H), 4.00–3.76 (m, 2H), 1.88–1.76 (m, 2H), 1.53–1.42 (m, 2H), 0.96 (t, *J* = 7.4 Hz, 3H). ^13^C NMR (101 MHz, CDCl_3_) δ: 145.42, 127.51, 127.27, 125.06, 122.42, 115.54, 47.20, 29.13, 20.29, 13.94.

10-octylphenothiazine (**PTZ-C8**).
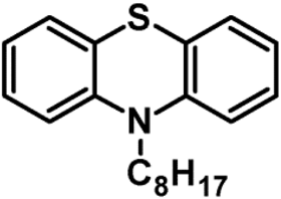


The crude product was purified by column chromatography (silica gel, hexane), to give **PTZ-C8** as a yellowish oil liquid (13.8 g, 88% yield). ^1^H NMR (400 MHz, CDCl_3_) δ: 7.22–7.11 (m, 4H), 7.02–6.81 (m, 4H), 4.00–3.62 (m, 2H), 1.90–1.76 (m, 2H), 1.49–1.40 (m, 2H), 1.37–1.21 (m, 8H), 0.89 (t, *J* = 6.8 Hz, 3H). ^13^C NMR (101 MHz, CDCl_3_) δ: 145.43, 127.50, 127.25, 125.05, 122.39, 115.50, 47.52, 31.88, 29.34, 29.23, 27.08, 27.03, 22.77, 14.25.

10-decylphenothiazine (**PTZ-C10**).
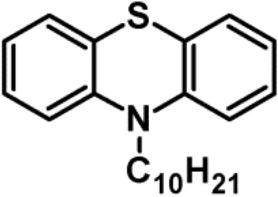


The crude product was purified by column chromatography (silica gel, hexane), to give **PTZ-C10** as a white waxy solid (12.4 g, 73% yield). ^1^H NMR (400 MHz, CDCl_3_) δ: 7.22–7.12 (m, 4H), 7.03–6.88 (m, 4H), 4.01—3.67 (m, 2H), 1.88–1.75 (m, 2H), 1.50–1.38 (m, 2H), 1.36–1.19 (m, 12H), 0.90 (t, *J* = 6.8 Hz, 3H). ^13^C NMR (101 MHz, CDCl_3_) δ: 145.59, 127.66, 127.43, 125.31, 122.57, 115.70, 47.66, 32.35, 29.99, 29.94, 29.75, 29.66, 27.29, 27.22, 23.15, 14.63.

### X-ray diffraction (XRD)

X-ray diffraction measurements were performed on a Rigaku Denki D/Max Rapid II diffractometer equipped with a rotating Ag anode, a graphite (002) monochromator, and an imaging plate detector in the Debye–Scherrer geometry. The wavelength of the *K*_*α*_ incident beam, $\lambda$, was 0.5608 Å. The samples were measured in the liquid state, in borosilicate glass capillaries, at room temperature. After subtraction of the background from the empty capillary, the diffraction intensity measured as the function of the scattering angle, 2$\theta$, was transferred to the function of the scattering vector, $Q=\frac{4\pi sin\theta }{\lambda }$, and normalized to the amplitude of the main diffraction peak.

### Broadband dielectric spectroscopy (BDS)

BDS measurements were performed using a Novocontrol GMBH Alfa analyzer in a wide frequency, *f*, and temperature, *T*, ranges. The temperature was controlled with high accuracy of 0.1 K by a Quatro controller using a nitrogen gas cryostat. The investigated glass-forming liquids were placed between steel electrodes of the capacitor with a 15 mm diameter. The fixed distance between electrodes (0.1 mm) was provided by fused silica spacer fibers.

## Results and discussion

### Molecular structure of investigated PTZ derivatives

**Figure 1:**
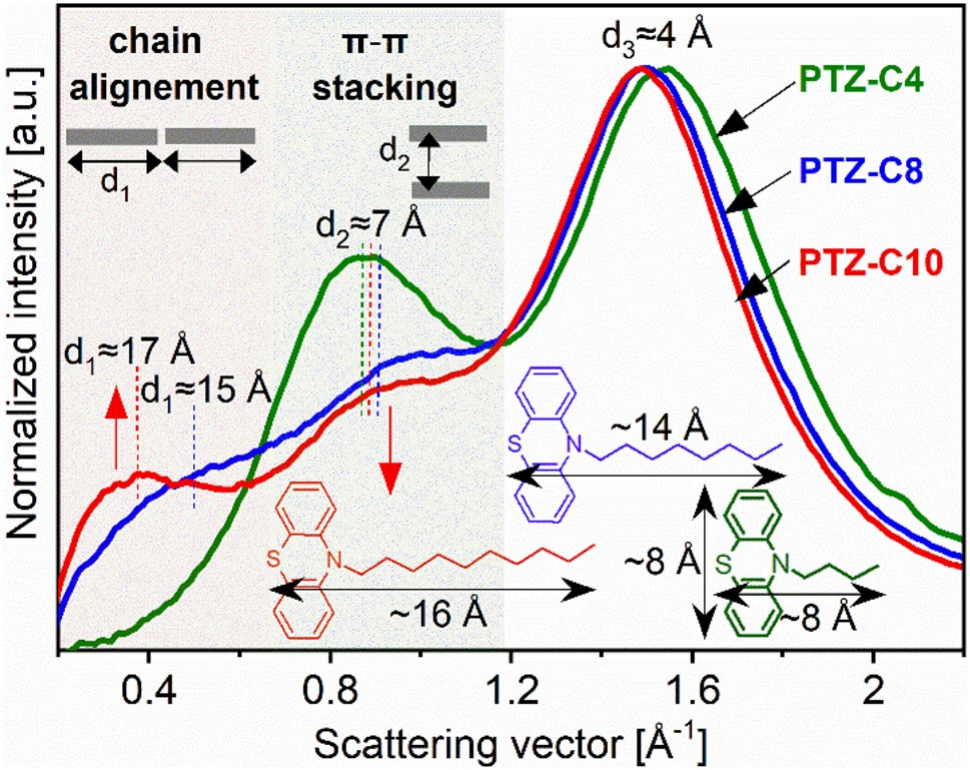
Comparison of the XRD patterns for PTZ-C4, PTZ-C8, and PTZ-C10 in the low-scattering vector range arising mainly due to the intermolecular structure. The presence of two pre-peak features for PTZ-C10 is the fingerprint of two types of intermolecular ordering, parallel π*–*π stacking of cores and longitudinal organization of chains. The average dimensions of molecules and intermolecular periodicities calculated from the positions of the diffraction peaks were indicated.

In the first step towards understanding the impact of self-organizing structural properties of PTZ derivatives on their dynamics, the XRD studies were performed. The analysis of X-ray diffraction patterns showed that modification of the alkyl chain length leads to a different organization of the intermolecular structure of the studied systems. For all PTZs, the registered diffraction patterns, shown in Fig. 1, are different from those observed for materials devoid of specific interactions and nanoscale uniform, described herein as ‘simple’ liquids. For PTZ-C4 one can see that except for the main diffraction peak arising at $Q$ ≈ 1.5 Å^−1^ due to the nearest-neighbor intermolecular correlations, also a strong pre-peak at $Q$ ≈ 0.9 Å^−1^ appears. The position of this peak corresponds, via the formula $Q=2\pi /d$, to a real-space periodicity, $d$, of around 7 Å. It must be stressed that phenothiazine-based molecules are known in the literature as systems with a strong tendency to form parallel stacked arrangements through π–π interactions between aromatic cores^8–10^. It is known that the intermolecular distance in the stacks depends on functional groups attached to the aromatic core. Theoretical calculations for unmodified PTZ molecules have demonstrated that in optimized models of stable dimers the intermolecular distance associated with the π–π stacking is ~ 6 Å^10^. Here, we observe a slightly greater distance between the stacking molecules, which may be affected by the presence of the alkyl chains attached to the aromatic core, but it hardly changes with increasing the chain length. The coherence length, $L$, associated with the range of order in the direction perpendicular to the stacking molecules, calculated by the formula $L=2\pi /w$ (where $w$ is the peak full width at half maximum), is ~ 18 Å. Such a value suggests the presence of small supramolecular clusters in PTZ-C4, composed of on average 3–4 π–π stacked molecules. It is in agreement with predictions that PTZ and its derivatives are good candidates for self-assembling in monomolecular layers^10^. Analyzing the pre-peak at ~ 0.9 Å^−1^ as we increase the alkyl chain, one can observe the decrease in the intensity and broadening of this diffraction feature. Such behavior indicates the destructive effect of the increasing chain length on the π–π stacking organization of molecules. The PTZ-C8 and PTZ-C10 systems are characterized by a considerably lower degree of this kind of order compared to PTZ-C4. Longer chains enhance the complexity of the supramolecular architecture and may involve distortions in the π–π stacking order. Interestingly, in the case of PTZ-C8, an additional diffraction bump appears at low $Q$ ≈ 0.5 Å^−1^. In turn, for PTZ-C10 one can see a pronounced pre-peak shifted even more towards lower $Q$. This proves the formation of the medium-range order of another nature than the π–π stacking for PTZ derivatives with *n* ≥ 8, at greater intermolecular distances. One should realize that the real-space periodicities related to the positions of the diffraction features at very low $Q$, 15 and 17 Å for PTZ-C8 and PTZ-C10, respectively, nearly correspond to the lengths of the molecules along their long axis, 14 and 16 Å for PTZ-C8 and PTZ-C10, respectively. This suggests an ordering of the PTZ molecules along their long axis for these PTZ derivatives. It seems, therefore, that there is a minimum alkyl chain length (here for *n* = 8) that is sufficient to drive such molecular self-aggregation at larger periodicities than the π–π stacking. Such effect was observed in some ionic liquids where the segregated domains formed by charged head groups and apolar alkyl chains are responsible for their locally-layered nanoscale structure^12–14^. The X-ray measurements performed on a homologous series of a model room-temperature ionic liquid (RTIL), [C_*n*_mim][NTf_2_], by Haddad et al. revealed that for *n* < 6 a typical liquid-like electron density profile was observed while for 8 < *n* < 20 the ordering of the polar and apolar moieties within respective layers gradually appeared^15^. Besides, the formation of segregated structures involving apolar alkyl chains with variable length have also been reported for polymers (*e.g.* poly(n-alkyl methacrylates)^16^, poly(n-alkyl acrylates)^17^, poly(di-n-alkyl itaconates)^18^, poly(alkylene oxides)^19^).

Based on the obtained results it is reasonable to assume that the self-assembly of the studied PTZ-based molecules is controlled by a balance between π*–*π stacking interactions of PTZ rings and interactions between alkyl chains. It is worth reminding that Bende et al*.* performed a theoretical investigation of intermolecular π–π stacking interactions between some PTZ derivatives with attached alkane chains varying from propane to decane^10^. Their calculations for selected dimer and pentamer configurations revealed that the pentane chain has the optimal length to facilitate the parallel aggregation of the aromatic rings. For longer chains, the appearance of the distorted oligomers and a destructive effect on the π–π stacking order was observed, which is in agreement with our experimental data.

### Molecular dynamics of PTZ derivatives in the supercooled liquid state

**Figure 2:**
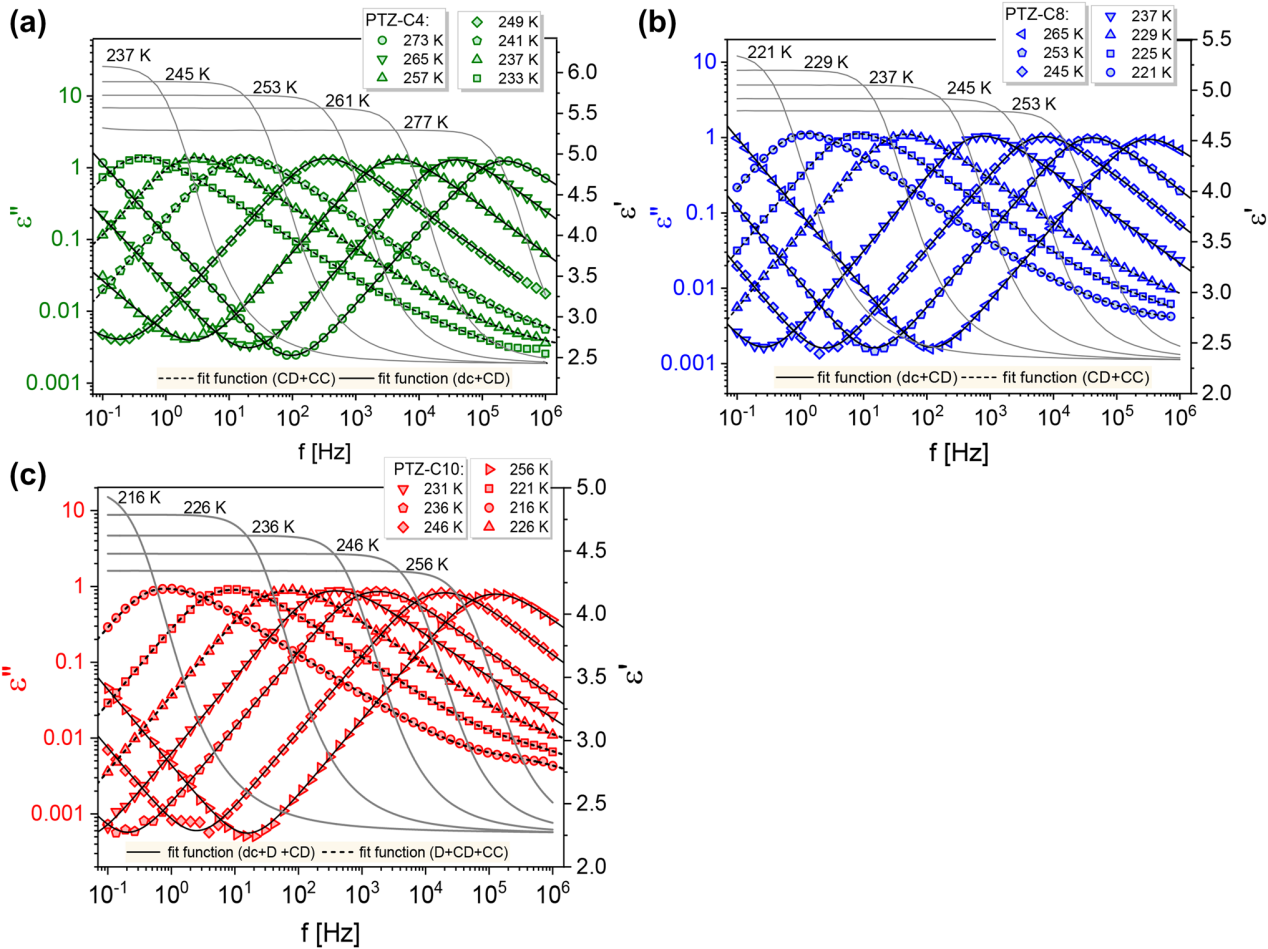
Grey lines and symbols correspond to experimental data showing real and imaginary parts of permittivity spectra registered for PTZ-C4 (**a**), PTZ-C8 (**b**), and PTZ-C10 (**c**) at selected temperatures. For dielectric loss data *ε*″(*f*) the fitting functions are also depicted as black solid and dashed lines. At lower temperatures, where a noticeable excess contribution is visible on the high-frequency slope of *α*-peak, the combination of fitting functions included the CC term was applied (shown as dashed lines).

To gain an insight into the relaxation dynamics of the series of PTZ derivatives, we measured the complex dielectric permittivity ε*(*f*) = *ε*′ (*f*) − *iε*″ (*f*). The real and imaginary parts of the spectra at selected temperatures above the glass transition temperature, *T*_*g*_, are presented in Fig. 2. With increasing frequency, *f*, the real part, *ε*′(*f*), decreases step-like. In contrast, the imaginary part, *ε*″(*f*), reveals the maximum usually attributed to the α-relaxation peak, genuine to any molecular liquid. This broad peak, spreading over several decades in frequency, shifts towards lower *f* on cooling, manifesting pronounced changes in the system dynamics. Its description with an appropriate fitting function was a starting point for further discussion.

**Figure 3:**
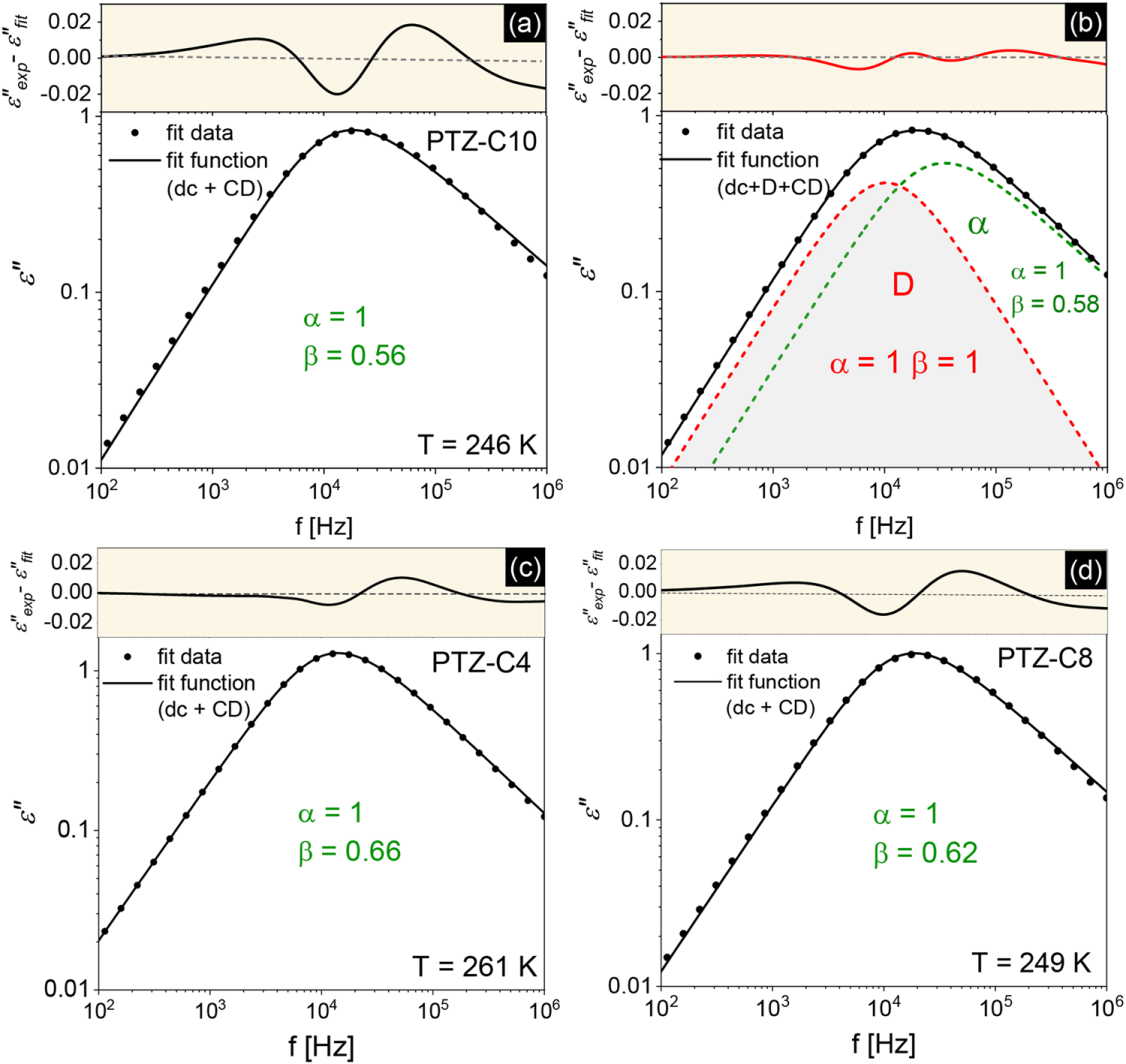
(**a**, **b**) The analysis of dielectric loss spectra for PTZ-C10 at *T* = 246 K for different fit strategies. Experimental data points are depicted as circles. The solid line is a resultant fit function, while the dashed line shows the fitting components. The values of shape parameters of the HN function (Eq. 1) are indicated. The upper panels show the differences between the experimental data points and fitted values. For comparison, the effect of single-function analysis for PTZ-C4 (**c**) and PTZ-C8 (**d**) is presented as well.

To parametrize the *ε*″* (f*) data, we used different combinations of empirical model functions with the dc-conductivity term, including asymmetric Cole–Davidson (CD), symmetric Cole–Cole (CC)^20^, and single-exponential Debye (D) functions^21^. The adopted fitting function can be expressed by the general Havriliak–Negami (HN) equation^22^:

1 $${\varepsilon }^{*}\left(\omega \right)={\varepsilon }_{\infty }+\sum_{i=1}^{n}\frac{\Delta \varepsilon }{{[1+{(i\omega {\tau }_{HN})}^{\alpha }]}^{\beta }}+\frac{{\sigma }_{dc}}{{\varepsilon }_{0}i\omega }$$

where *ω* = 2*πf* is an angular frequency, *ε*_∞_ is the dielectric constant in the high-frequency limit, *Δε* is the relaxation strength of the respective process, and *τ*_NH_ is a characteristic HN-relaxation time. Exponents *α* and *β* are shape-parameters. The HN formula translates into Debye function with *α* = *β* = 1, CD function with *α* = 1 and 0 < *β* < 1, and CC function with 0 < *α* < 1 and *β* = 1. It is worth mentioning that in all tested PTZs derivatives, the conductivity contribution was well-separated from the main peak in the whole range of tested temperatures. We will start the discussion from the results obtained for the PTZ derivative with the longest chain attached (PTZ-C10). Although at first glance, on the dielectric loss spectrum for PTZ-C10 in Fig. 2c, a single relaxation peak is visible, its parameterization with a single CD function (with low-frequency dc-conductivity term, according to Eq. 1) did not give a satisfactory outcome. The results of analyzes carried out with different fit strategies are presented in Fig. 3a,b.

Regardless of the considered frequency range (the narrower *f* range was taken into account, the smaller the *β* value in CD function), the level of matching of experimental data and the single fitting function in the case of PTZ-C10 was insufficient. The appropriate description of experimental data was possible only when a combination of the Debye and CD functions was applied. Such an approach has the serious disadvantage of making multiple assumptions about the shape parameters. It is especially inconvenient when the analysis concerns several poorly resolved spectral contributions. In some cases, better peak separation can be obtained by derivative analysis allowing to approximate the peak position from the real part of the dielectric permittivity data^23^. However, for PTZ-C10, this proved to be ineffective. The upper panels in Fig. 3a,b show the differences between the experimental data and the fit data for applied methods of analysis. It is clear that the differences were much smaller when a combination of two fitting functions was used (Fig. 3b).

For most glass-forming liquids, the structural relaxation dominates the spectrum of dielectric losses in the supercooled liquid state. In contrast, a sub-group of liquids with the ability to self-organization can be given, in which an additional slow Debye peak may appear^24–28^. Its emergence is usually accompanied by the presence of a so-called ‘pre-peak’ in the diffraction pattern before the main amorphous halo ubiquitous for ordinary liquids^29^. However, our knowledge of the relationships between the features of the supramolecular organization seen by the X-ray and neutron diffraction methods and the slow relaxation processes probed by the dielectric spectroscopy is still limited^30^. For instance, there are materials displaying the Debye-like relaxation on dielectric loss spectra despite no direct fingerprints of the medium-range intermolecular order in the diffraction data. Although such examples are rare (e.g. water^31^, 4-phenyl-1-butanol^32^) they show that the relationship between the presence of slow Debye relaxation in dielectric response and mentioned structural features is not universal.

**Figure 4:**
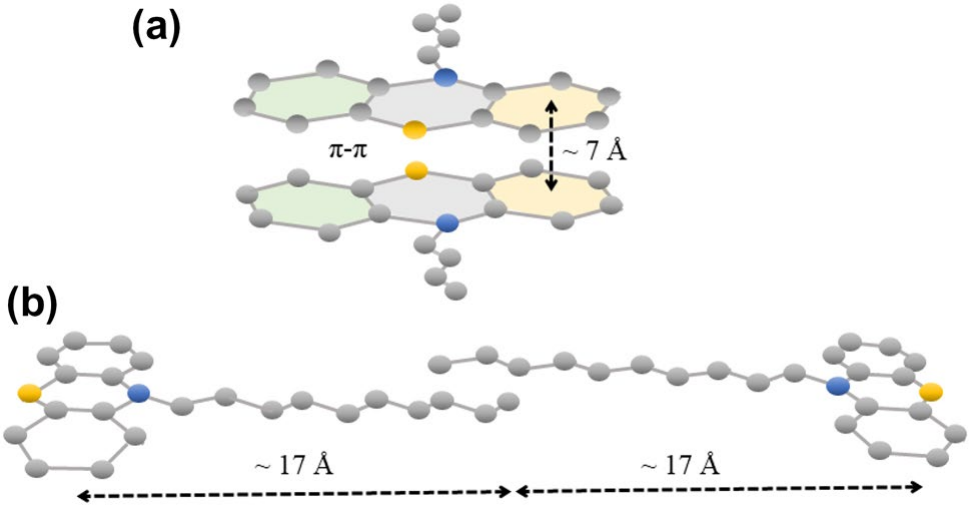
Scheme of a possible arrangement of molecules in PTZ-C4 (**a**) and PTZ-C10 (**b**).

To rationalize the atypical shape of the main peak in PTZ-C10 (that cannot be parametrized well by a single CD function) the presence of additional slow Debye relaxation was postulated. Taking into account the results of XRD measurements, one possibility is its interpretation as a dynamic signature of nanoscale aggregates. Considering the possible mechanism of eventual slow relaxation in PTZ-C10, the most adequate model we can propose is a linear cluster formed by oppositely directed PTZ molecules shown in Fig. 4b. Such an arrangement depends on the chain length (so the cluster will be the most extensive for PTZ-C10), but also allows for π–π interactions between aromatic cores, thus is consistent with structural data. The mechanism of apparent relaxation could be analogous to the transient chain model proposed for monohydroxy alcohols and results from the change in the dipole moment accompanying the detachment and attachment of molecules to the opposite ends of the elongated molecular cluster^33^.

Figure 3c,d shows the results of the fitting procedure performed for PTZ-C4 and PTZ-C8. Interestingly, in these cases applying a single CD function with the dc-conductivity yielded a better parameterization of the *α*-relaxation region. For PTZ-C4 no indication of the presence of additional spectral contribution was found. For PTZ-C8 some deviation between experimental and fitting data can be noticed, but not as substantial as in PTZ-C10. Since the situation in PTZ-C8 can be considered unclear, we will focus on PTZs with the shortest and the longest chains, for which the discrepancies in dielectric and XRD diffraction patterns were undeniable. The results in Fig. 3c indicate that for PTZ-C4 the dynamics of parallel stacked arrangements through π–π interactions (see example shown in Fig. 4a) are not manifested in the dielectric loss spectra. The atypical broadening of the main relaxation peak is observed only when PTZ molecules self-assemble along the long axis. There may be several reasons why the dynamics of small clusters in PTZ-C4 were not detected. The first is very trivial and related to the stability of structures formed. The lifetime of aggregate may be shorter than the time required for its reorganization. Then, the structure breaks down before the dipole has time to reorient. Another explanation may be the non-polar nature of an aggregate created by an even number of molecules with opposite dipole moments. The coherence length of ~ 18 Å found for PTZ-C4 in the direction perpendicular to the stacking molecules suggests that 3–4 π–π stacked molecules can form a cluster. The lack of contribution to the dielectric response suggests that the tetrameric aggregates might prevail in the system.

**Figure 5:**
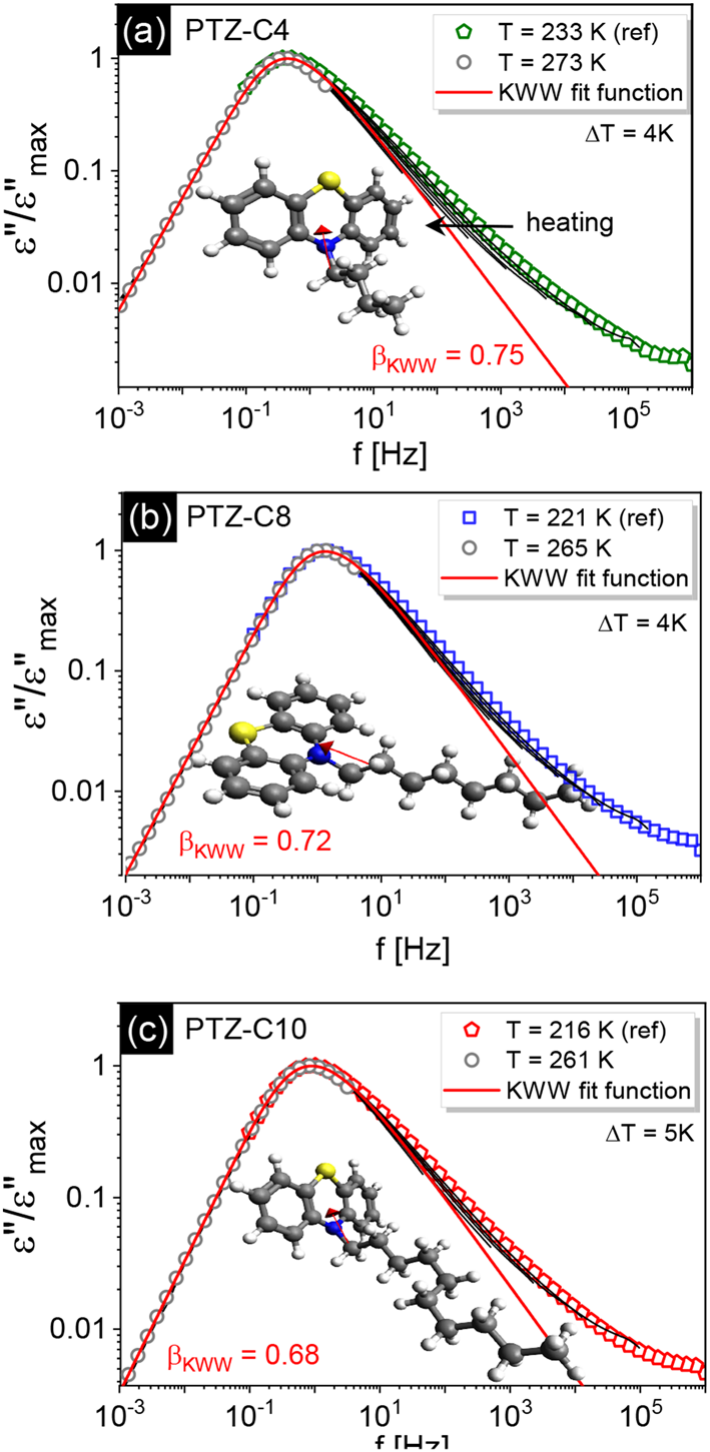
Master curves created by horizontal shifting of dielectric loss spectra on the reference spectrum (the spectra recorded for the highest and the lowest temperatures are shown as symbols, while those corresponding to the middle temperatures are depicted as black lines). The KWW fits to the dielectric loss spectra at reference temperatures are shown (*β*_*KWW*_ indicated). For PTZ-C10 total dielectric response was included in the analysis. The inserts show the chemical structure of the molecules along with the direction of the resultant dipole moment vector (red arrow).

The molecular arrangement proposed for PTZs with shorter and longer alkyl chains assumes the anti-parallel dipole alignment. This is in line with calculated values of the Kirkwood correlation factor, *g*_*K*_, expressing static correlations between interacting dipoles. For the determination of *g*_*K*_ we used the following formula^21^:

2 $${g}_{K}=\frac{9{k}_{B}{\varepsilon }_{0}MT\left({\varepsilon }_{s}-{\varepsilon }_{\infty }\right)\left({2\varepsilon }_{s}+{\varepsilon }_{\infty }\right)}{\rho {N}_{A}{\mu }^{2}{\varepsilon }_{s}{\left({\varepsilon }_{\infty }+2\right)}^{2}}$$

where *k*_*B*_ is the Boltzmann’s constant, *M* is molar mass, *ρ* is density, *N*_*A*_ is Avogadro’s number, *μ* is molecular dipole moment. The direction of dipole moment for investigated PTZ molecules is sketched in Fig. 5. In all PTZ derivatives, the dipole moment is oriented parallel to the alkyl chain, making the nitrogen atom the positive end of a dipole. The values of the dipole moment were determined for optimized structures of PTZs ensuring the minimum energy. In our calculations, the geometry optimization was performed using the B3LYP/6-31G** database (Becke 3-therms Lee, Young, Parr hybrid functional; with 6-31G** Gaussian functional basis). The computed values of dipole moment *μ* = 2.125 D for PTZ-C4, *μ* = 2.175 D for PTZ-C8, *μ* = 2.194 D for PTZ-C10, show a very slight dependence on the alkyl chain length. Therefore, regardless of changes in the chemical structure, the probe (dipole) providing information about the sample dynamics is similar in the systems under consideration. The densities of investigated PTZs in the temperature range corresponding to the supercooled liquid state were obtained by a linear extrapolation of higher-temperature values (see Supplementary materials for more details). For non-associating systems, in which the dipole moments of individual molecules are not correlated with each other, *g*_*K*_ = 1. In other cases, the value of *g*_*K*_ can be greater or smaller than 1, depending on whether the dipole moments of the molecules are arranged in a parallel or anti-parallel manner^34^. Deviations from *g*_*K*_ = 1 were found for all PTZ derivatives. Using the permittivity data shown in Fig. 2 we calculated *g*_*K*_ (*T* = 237 K) = 0.84 for PTZ-C4, *g*_*K*_ (*T* = 225 K) = 0.83 for PTZ-C8 and *g*_*K*_ (*T* = 221 K) = 0.82 for PTZ-C10. The obtained values are modest below unity and indicate that the molecular organization favors the anti-parallel arrangement of the dipole moments.

The alternative explanation for the peculiar spectral shape of loss peak in PTZ-C10 is related to the specific molecular composition with aromatic core and alkyl side chain that can give rise to the dynamical heterogeneity. The unusual broadening may be due to the separation of time scales of two distinct dynamics involving these distinct structural compartments. Such bimodal behavior related to the two-component nature of ionic liquids was reported recently^35^. It was also reported for poly(alkylene oxide)s where the *α*-peak of systems with side chains containing at least four carbon atoms show an atypical shoulder at high frequencies with a temperature-dependent shape^30^. Among various common features characterizing poly(alkylene oxide)s and PTZs investigated herein, one significant difference can be found. As presented in Fig. 5 in PTZs a narrowing of the *α*-peak with increasing temperature was observed. If the unusual peak shape in PTZ-C10 is due to the overlapping of *α*-process dynamics and the dynamics of the non-polar side chains coupled to the polar PTZ-based core, we would expect that at high temperatures, where the degree of heterogeneity is lower, the individual character of both processes will be more exposed. This is the case observed for poly(alkylene oxide)s where a systematic broadening of the α-relaxation was found with increasing temperature, including the appearance of a double-peak structure at the highest temperature^36^. The distinct behavior found for PTZ-C10 makes the first scenario related to the higher-order molecular organization more likely.

The spectral shape of the structural relaxation received a lot of attention recently^1,2,37^. One of the topics that have been widely discussed is the generic line shape of the structural relaxation (following ∝ *ω*^−1/2^ at high frequencies) and possible deviations from such universal behavior due to the contribution of cross-correlations among various dipole moments^38^, as indicated by the recent theory of Déjardin et al.^39^. It was evidenced that slow Debye mode in the dielectric response of some materials (e.g. 1-propanol^40^, glycerol^41^) may be due to additional contribution originated from the cross-correlation part of the response function. Based on this idea, the occurrence of Debye relaxation was rationalized for polar liquid tributyl phosphate lacking hydrogen-bonding interactions^42^. According to the theory of Déjardin et al. which supports the quoted concept, an additional process in the dynamic susceptibility will appear only if the *g*_*K*_ factor exceeds unity^39^. Thus, in a line with this, no additional process due to dipole–dipole interactions should appear in the case of PTZs (*g*_*K*_ < 1).

**Figure 6:**
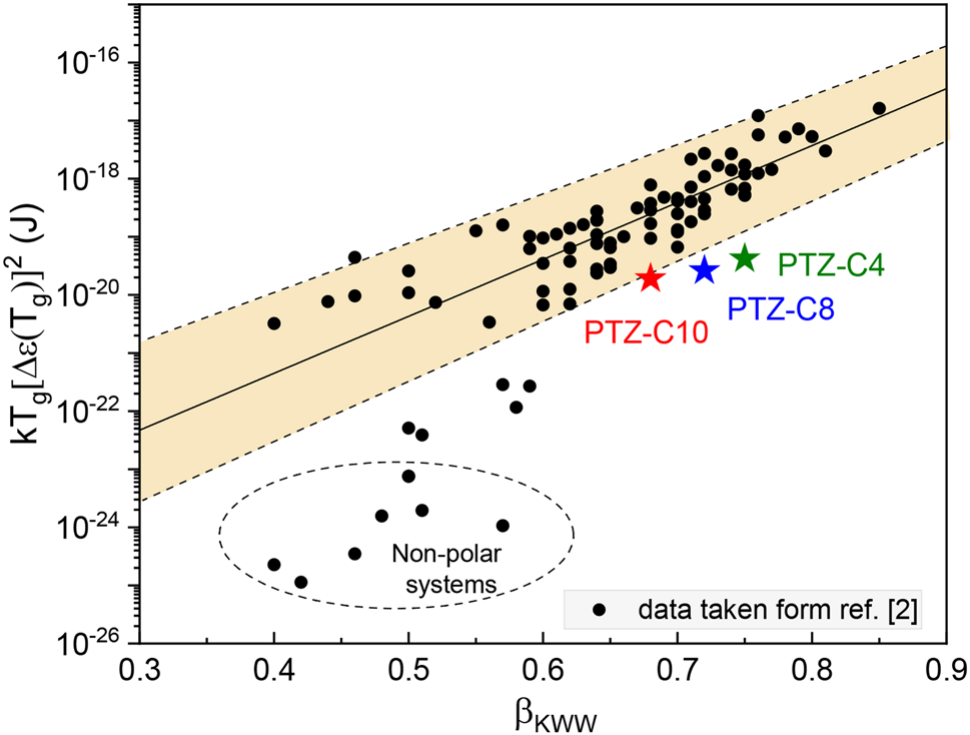
The correlation between *kT*_*g*_[Δ*ε*(*T*_*g*_)]^2^ and *β*_KWW_, a stretching parameter of KWW function^2^. Reprinted with permission from Paluch et al.^2^. Copyright 2016 by the American Physical Society. Data for investigated PTZs have been added (marked as stars), showing that they are not in line with the general trend.

Before we move on to the discussion on the temperature evolution of the parameters describing the dynamics of PTZ derivatives, we will return to the data presented in Fig. 2. The *ε*′(*f*) data show the high- (ε_∞_) and low-frequency (ε_s_) plateaus in the real part of complex permittivity, which was used to calculate the dielectric strength, Δ*ε* = ε_s_ − ε_∞_. Comparing the values of Δ*ε* near *T*_*g*_, i.e., Δ*ε* (*T* = 237 K) = 3.7 for PTZ-C4, Δ*ε* (*T* = 225 K) = 3.0 for PTZ-C8, Δ*ε* (*T* = 221 K) = 2.6 for PTZ-C10, their decrease with increasing chain length was found. It is generally accepted that the higher the value of dielectric strength, the narrower the structural relaxation peak^2^. According to this rule, the narrowest distribution of relaxation time is expected for PTZ-C4. Such a trend was illustrated by Paluch et al. for many glass-forming liquids, classified mainly as van der Waals liquids^2^. Although the examined systems are not typical ‘simple’ liquids, it is worth checking whether this relationship is correct. The dielectric loss spectra registered in the vicinity of the glass transition were fitted using the one-side Fourier transform of Kohlrausch–Williams–Watts (KWW) function *ψ* (*t*) = exp [− (*t*/*τ*_*α*_)^*β*^_KWW_] with stretching parameter *β*_KWW_. The analysis yielded *β*_KWW_ (*T* = 233 K) = 0.75 for PTZ-C4, *β*_KWW_ (*T* = 221 K) = 0.72 for PTZ-C8, and *β*_KWW_ (*T* = 216 K) = 0.68 for PTZ-C10. Master curves in Fig. 5 show that for all systems the time–temperature superposition is invalid. With increasing *T*, we observed a slight narrowing of the *α*-loss peak for each system and a related increase in *β*_KWW_ values. At *T* = 273 K *β*_KWW_ = 0.83 for PTZ-C4, for PTZ-C8 *β*_KWW_ = 0.80 at *T* = 265 K, whole for PTZ-C10 *β*_KWW_ = 0.75 at *T* = 256 K. Figure 6 shows the mentioned correlation with the added data for PTZ-derivatives indicating that the trend between Δ*ε* and *β*_KWW_ observed for many glass-forming liquids is not satisfied by these materials^2^. The intuitive explanation is that contrary to van der Waals liquids, the dipole–dipole interactions are not the main factor determining their behavior. In this case, the presence of aromatic units acting as the driving force for the self-organization process seems to gain more importance.

To complete the discussion on the dynamics of PTZ derivatives in the supercooled liquid state, we calculated the most probable relaxation time as structural relaxation time, *τ*_*α*_, and compared its temperature dependence for all investigated systems. The applied relation between *τ*_*α*_ and the fitting parameters of the HN function is given below^21^:

3 $${\tau }_{\alpha }={\tau }_{HN}{\left[sin\left(\frac{\pi \alpha }{2+2\beta }\right)\right]}^{-1/\alpha }{\left[sin\left(\frac{\pi \alpha \beta }{2+2\beta }\right)\right]}^{1/\alpha }$$

**Figure 7:**
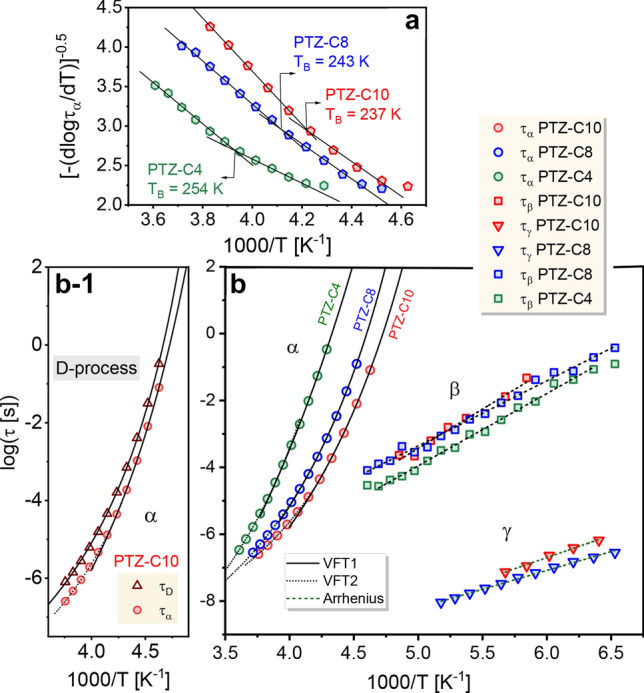
(**a**) The derivative analysis of the temperature dependence of structural relaxation times for PTZ derivatives. The crossover temperature *T*_*B*_ was determined from the intersection of two straight lines in the low- and high-temperature regime. (**b**) A plot of log(*τ*) versus the reciprocal of temperature for the respective relaxation times (circles correspond to *α*-process, squares to *β*-process, triangles to *γ*-process). (**b-1**) The temperature evolution of relaxation times for slow and fast components according to the indication in Fig. 3b. The solid and dot lines denote the VFT fit function in the low and high-temperature regime according to the indication of the Stickel analysis; the dashed lines are the Arrhenius fits. The corresponding fitting parameters are summarized in Tables 1 and 2.

Figure 7b shows a plot of log(*τ*_*α*_) versus 1000/T for all investigated PTZ derivatives. In Fig. 7b-1 the temperature dependencies of characteristic relaxation times for slow Debye-mode and α-process are depicted for PTZ-C10. We describe this data using the Vogel–Fulcher–Tammann (VFT) fit function, *τ*_*α*_ (*T*) = *τ*_0_ exp[*D T*_0_/(*T* − *T*_0_)] with *τ*_0_, *D*, and *T*_0_ as fit parameters^43–45^. To determine the *T*_*g*_ value, the VFT fit was extrapolated to the low temperatures where the structural relaxation time *τ*_*α*_(*T*_*g*_) = 100 s. In the case of PTZ-C4, we noted that fitting the data with a single VFT function led to a physically unaccepted value of the pre-exponential factor ~ 10^–16^ s. As practice shows, it suggests that in the investigated temperature range, two VFT fitting functions may be necessary to describe the temperature evolution of *τ*_*α*_ with physically intelligible parameters^46^. To properly define the fit limits, we used a derivative analysis proposed by Stickel et al*.*^47^ The results of this analysis are presented in Fig. 7a. Indeed, in the case of PTZ-C4, two temperature regions, indicated by the intersection of two straight lines at crossover temperature *T*_*B*_ = 254 K, are clearly visible. Such behavior indicates the quantitative changes in the molecular dynamics in these regions, the description of which may require a separate set of VFT parameters. For the two remaining liquids, also two linear regions were distinguished in Fig. 7a with the intersection at *T*_*B*_ = 243 K for PTZ-C8 and *T*_*B*_ = 237 K for PTZ-C10. Fitting the data according to the indications of Stickel’s analysis resulted in two VFT functions with fitting parameters summarized in Table 1. Such dynamic crossover has been observed for various glass-forming liquids, usually at *T* corresponding to 1.1–1.6 *T*_*g*_^48^. In investigated PTZs, it is observed at temperatures of ~ 1.14 *T*_*g*_ for PTZ-C4 and ~ 1.15 *T*_*g*_ for PTZ-C8 and PTZ-C10. Such behavior can be rationalized by the presence of intermolecular interactions, which variously affect the dynamics of PTZs in regions of low and high temperatures^46^.

**Table 1:** The summary of the discussed parameters characterizing the dynamics of PTZ derivatives above *T*_*g*_: VFT fitting parameters (± standard error), glass transition temperatures and fragilities (± uncertainty value) calculated from VFT fit data, glass transition temperatures determined from DSC.

BDS							DSC
PTZ	*T* regime	VFT fitting parameters			*T*_*g*_ (K) (for *τ*_*α*_ = 100 s)	*m*	*T*_g_^*DSC*^ (K)
		log *τ*_*0*_	*D*	*T*_*0*_ (K)			
***α*-process**							
C4	Low	− 19.7 ± 0.7	28.5 ± 3.2	141.9 ± 4.1	222.8 ± 9.4	58 ± 5	227.68
	High	− 13.5 ± 0.2	7.7 ± 0.4	187.6 ± 1.8			
C8	Low	− 13.4 ± 0.5	9.6 ± 1.0	165.6 ± 2.7	210.7 ± 5.0	72 ± 4	213.29
	High	− 12.3 ± 0.1	7.3 ± 0.1	173.0 ± 0.7			
C10	Low	− 13.6 ± 0.6	10.2 ± 1.4	159.6 ± 3.6	205.0 ± 6.5	71 ± 5	209.17
	High	− 10.8 ± 0.1	4.7 ± 0.2	179.0 ± 1.4			
**D-process**							
C10		− 11.1 ± 0.2	6.2 ± 0.3	172.4 ± 1.4	208.0 ± 2.1	76 ± 2	

The determined values of *T*_*g*_s are presented in Table 1. They agree within a few degrees with those determined by the differential scanning calorimetry (DSC) studies (see Supporting materials for more details). The presence of a flexible alkyl side chain attached to a stiffer tricyclic core containing two aromatic benzene rings has a significant impact on the observed *T*_*g*_ values, leading to their decrease with increasing chain length (internal plasticization effect). In studies of molecular glasses with related chemical structures but with different molecular weights, an increase in *T*_*g*_ is usually observed in larger glass-formers^49,50^. Our previous studies on glass-formers with the structure based on 4-methyl-1,3-dioxolan backbone indicated a trend of an increase in *T*_*g*_ along with the elongation of the alkyl chain^28^. The effect observed here in the PTZs derivatives is analogous to that observed in some polymeric glass-formers, where the end group of the polymer introduces an additional free volume, reducing the observed *T*_*g*_ value. Wuest and Lebel observed a similar trend in *T*_*g*_ values for several structurally related non-polymeric 4,6-bis(mexylamino)-1,3,5-triazines containing alkyl chains of various lengths^51^. Taking into account the complexity of the molecular structure, this behavior is quite counterintuitive, as interactions between adjacent molecules are commonly regarded as constraints limiting the mobility of the system^52^. It is known that the presence of moieties involved in the intermolecular interactions usually increases *T*_*g*_^50^. However, such an effect was not observed for the investigated PTZs, and the PTZ-C10 derivative with the most complex molecular structure was characterized by the lowest *T*_*g*_ value.

To quantify the character of changes in the dynamics of the investigated glass-forming liquids close to *T*_*g*_, the fragility index, *m* = *d*log(*τ*_*α*_)/*d*(*T*_*g*_/*T*)|_*T*=*Tg*_, was determined from VFT fits^53^. While the bulkiness of the alkyl side chains and their mobility were found to be critical to the observed *T*_*g*_ values, it had slight impact on the fragility indices calculated for PTZs and summarized in Table 1. The fragility values for investigated systems are not substantially different and can be approximate by *m* ≈ 67 ± 8, hence according to Angell classification, they are intermediate-fragile glass-formers. The almost identical *m* values for PTZ-C8 and PTZ-C10 mean that the steepness of the temperature variations of structural relaxation near the glass transition is similar in both liquids. Such behavior was also reflected in the value of the *D* parameter (called fragility or strength parameter) in the VFT fitting function, which in the low-temperature regime was almost identical (D ≈ 10) for these systems. The lack of changes in material fragility was also observed in recent studies of Körber et al*.* on a series of high-*T*_*g*_ glass-formers based on non-polar 9,9′-spirobi[9H]fluorene core^54^. Regardless of the chemistry of the side substituents, the observed fragility was the same (*m* = 76). In a study on quinoline analogs, which contrary to glass-formers studied herein had a very similar bulkiness, Saini et al*.* shown that the most spectacular variations in the fragility (from *m* = 59 for quinoline contain phenyl rings to *m* = 131 for decahydroisoquinoline with non-aromatic cyclohexyl rings) were due to variations in the ring flexibility and stronger intermolecular interactions via the dipole–dipole interactions^55^. However, such behavior was found to be non-universal. In the polymeric glasses, the value of the fragility index reported for poly(phenylmethacrylate) is higher (*m* ~ 91) than that for poly(cyclohexylmethacrylate) (*m* ~ 71)^56^. These inconsistencies just prove that understanding the structural parameters that control fragility remains a challenge. Looking at our results from the perspective of the molecular structure of the studied systems, it can be concluded that the slightly smaller value of fragility index for PTZ-C4 in comparison to PTZ-C8 and PTZ-C10 may reflect a difference in the intermolecular organization evidenced by XRD studies.

### Molecular mobility of PTZ derivatives below ***T***_***g***_

**Figure 8:**
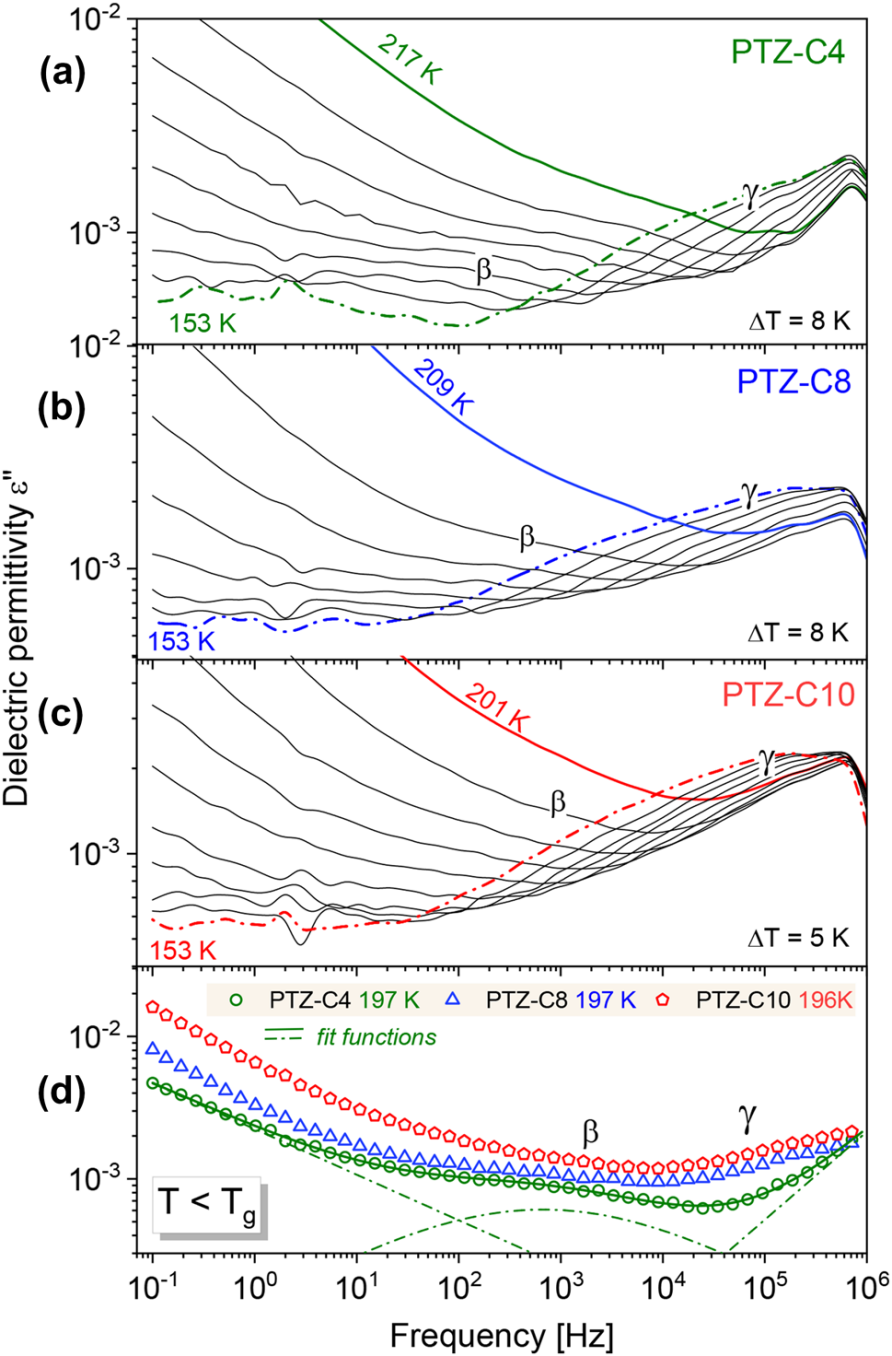
Representative dielectric loss spectra for PTZ derivatives collected below *T*_*g*_. The lowest panel shows a comparison of loss data at temperatures where *τ*_*β*_ is comparable. The solid line is the resultant fit function for PTZ-C4 at 197 K (superposition of appropriate HN functions). The dashed lines show the individual components of the fitting function.

In the glassy state, when the cooperative molecular motions are frozen due to extremely long time scales, the secondary modes prevail in the dielectric response. The dielectric loss spectra for PTZ derivatives measured below *T*_*g*_ are presented in Fig. 8. Two secondary relaxations can be distinguished for all systems. The first, located on the high-frequency flank of *α*-peak, is assigned as *β*-relaxation. The second, visible at higher frequencies, is referred to as *γ*-relaxation.

To parametrize the *ε*″ (*f*) data below *T*_*g*_ and to determine the relaxation times for each secondary mode, i.e., *τ*_*γ*_ and *τ*_*β*_, the different combinations of CC and CD functions were applied. Among the investigated PTZ derivatives, the *γ*-process for PTZ-C4 was the fastest, which makes the extraction of *τ*_*γ*_ impossible. The calculated relaxation times for *β*- and *γ*-processes revealed, typically to glassy matter, an Arrhenius-type temperature dependence, shown in Fig. 7b. The values of the activation energy barrier, *E*_*a*_, obtained by fitting the equation* τ* = *τ*_*∞*_ exp(*E*_*a*_/*RT*) to the experimental data in Fig. 7b, where *τ*_*∞*_ is the pre-exponential factor and *R* is the gas constant, are given in Table 2. From a general point of view, the molecular origin of the observed fast secondary relaxations in PTZ derivatives can be twofold. The observed processes may be the Johari–Goldstein (JG) relaxation reflecting the motions of all atoms in molecule or may result from the internal mobility of its fragment^57,58^. When we look at the chemical structure of the studied systems, the assumption immediately arises that one of them will reflect the mobility of the alkyl chains. The lower activation energy of *γ*-process in comparison to *β*-relaxation, i.e., *E*_*a*_ = 21.6 kJ/mol for PTZ-C8 and *E*_*a*_ = 25.6 kJ/mol for PTZ-C10 for *γ*-process, and *E*_*a*_ = 37.2 kJ/mol for PTZ-C8 and *E*_*a*_ = 44.9 kJ/mol for PTZ-C10 for *β*-process, supports the interpretation that the mechanism of the former is related to the fast rotation motion of side chains. Similar activation energy attributed to the rotation of the *n*-alkyl chains was reported by Musiał et al*.* for ionic liquids based on tricyanomethane-containing alkyl chains of different lengths^59^. In this study, the height of the energy barrier ascribed to the rotation of the alkyl chains ranged from 18 kJ/mol (for *n* = 4) to 25 kJ/mol (for *n* = 8). The growth of *E*_*a*_ with the increasing length of the alkyl substituent was explained by the growing steric hindrance, which impedes the motions in longer chains.

**Table 2:** The parameters obtained from the fitting of the *τ*_*β*_ and *τ*_*γ*_ data depicted in Fig. 7b to the Arrhenius equation.

	*β*-relaxation		*γ*-relaxation	
	log *τ*_*∞*_	*E*_*a*_ (kJ/mol)	log *τ*_*∞*_	*E*_*a*_ (kJ/mol)
PTZ-C4	− 14.8 ± 0.2	41.4 ± 0.8		
PTZ-C8	− 13.1 ± 0.3	37.2 ± 0.9	− 13.8 ± 0.1	21.6 ± 0.4
PTZ-C10	− 15.2 ± 0.8	44.9 ± 2.8	− 14.7 ± 0.4	25.6 ± 1.2

Due to the high stiffness of the phenothiazine core, we assumed that the possibility that *β*-relaxation is a manifestation of its mobility is less probable. Thus, the *β*-process was attributed to JG relaxation. Future high-pressure studies will verify such classification based on the pressure sensitivity criterion. Among the investigated PTZ derivatives the highest activation energy for the *β*-process was found in PTZ-C10, but on the other hand, in the presence of the assessed uncertainties, these differences are rather small. This can be explained when one takes into account the strong overlapping of *β*- and *γ*-processes leading to tentative errors during their deconvolution. This may explain only minor differences in the *β*-relaxation dynamics of these structurally different glasses. The impact of differences in the intermolecular arrangement on glassy dynamics is more pronounced in the case of mobility giving rise to the *γ*-process. Our results show that the rotation of the shortest side chain is much easier in the system with a limited intermolecular order. The lack of constraints in the form of a more complex network of interactions does not impede the chain rotation in PTZ-C4, leading to the shorter time-scale of *γ*-process in PTZ-C4, in comparison to the two remaining PTZ systems with longer side chains.

## Conclusion

In this article, we performed a detailed investigation of the impact of the differences in the molecular structures of PTZ-based glass-forming liquids on their dynamic behavior, demonstrating some structure-dynamics correlations and their lack. The chemical structure of the investigated materials with identical tricyclic cores was modified by the substitution of *n*-alkyl chains with different lengths (*n* = 4, 8, 10). As indicated by the results of the XRD studies, such structural modification differentiates the intermolecular organization of the investigated materials and their ability to form various supramolecular structures. Although the diffraction patterns for all three PTZ derivatives are distinct from those usually reported for non-interacting liquids, only in the case of PTZ-C10 to explain the atypical spectral shape, the presence of additional slow Debye relaxation was postulated. This extra process, described widespread as supramolecular relaxation, was practically invisible at first sight (no clear contribution on the low-frequency slope of *α*-peak is visible). However, the detailed analysis of dielectric data revealed atypical broadening near the maximum of prominent loss peak in *ε*″(*f*) data, not parameterizable by a single CD function. We interpreted this as a sign of the presence of an additional contribution to the dielectric spectrum, which we associated with the structural complexity of the PTZ-C10 nanostructure involving π*–*π stacking and ordering of molecules along their long axis, as suggested by the XRD studies. Interestingly, this behavior was undetectable for PTZ-C4, where the π*–*π stacking is preferred over the supramolecular organization of alkyl chains. It means that only in PTZ-C10 interactions between the molecules result in the structure able to contribute to the low-frequency Debye-like dynamics. For investigated PTZs, the increase in the medium-range order was manifested as a broadening of the dielectric loss peak reflected in the lower value of stretching parameter *β*_KWW_. Also, for all materials, a disagreement with the behavior observed for non-associating liquids was revealed as a deviation from the anti-correlation between the value of the stretching parameter *β*_KWW_ and the relaxation strength of the *α*-process. Apart from that, our study revealed some interesting dynamics-structure relationships. With the side chain extension, we observed the decreasing of *T*_*g*_ and only a minor difference in the fragility. The molecular interactions are often perceived as factors constraining the collective dynamics and increasing the *T*_*g*_ values. Meanwhile, in these systems based on a rigid tricyclic core linked covalently with flexible chains, the length of side chains has a decisive impact on the properties, exerting a plasticizing effect on the dynamics. In the glassy state, there were also noticeable differences in the dynamics of these structurally related glass-formers. The highest activation energy of secondary processes was observed for the derivative with the longest alkyl chain, where the spatial constraints imposed by molecular interactions were the most relevant.

Understanding how the molecular structure impacts the dynamics of glass-forming liquids has been a longstanding question that still requires clarification. The presence of interactions between molecules that may result in diverse molecular associations, organizations, and medium-range ordering, complicate our comprehension of their dynamics, leading to the emergence of various phenomena arising from structural complexity. We hope that our study will stimulate further research efforts towards the more systematic work needed to link the details of the molecular structure with the parameters that describe the dynamics of glass-forming systems.

## Supplementary Information

Supplementary Information.
