# Supplementary material for: The dielectric response of phenothiazine-based glass-formers with different molecular complexity
Source: Sci Rep. 2021 Aug 4;11:15816. doi: 10.1038/s41598-021-95127-y (PMC8338989; doi:10.1038/s41598-021-95127-y)
Supplement: Supplementary file 1 — Supplementary Information. [file 41598_2021_95127_MOESM1_ESM.docx]

**Supplementary Information**

**The dielectric response of phenothiazine-based glass-formers with different molecular complexity**

M. Rams-Baron,^1,2,*^ A. Jędrzejowska,^3^ K. Jurkiewicz,^1,2^ M. Matussek,^4^ M. Musiał^1,2^,
M. Paluch^1,2^

*^1^August Chełkowski Institute of Physics, University of Silesia in Katowice, 75 Pułku Piechoty 1, 41-500 Chorzów, Poland*

*^2^Silesian Center for Education and Interdisciplinary Research, 75 Pułku Piechoty 1a, 41-500*

*Chorzów, Poland*

*^3^PET Diagnostics Department, Maria Sklodowska-Curie Memorial Cancer Centre and Institute of Oncology, Gliwice Branch, Poland*

*^4^Institute of Chemistry, University of Silesia in Katowice, Szkolna 9, 40-006 Katowice, Poland*

*corresponding author: [marzena.rams-baron@us.edu.pl](mailto:marzena.rams-baron@us.edu.pl)

Table S1. Parameters used to estimate the value of Kirkwood correlation factor for investigated systems.

| Parameters | **PTZ-C4** | | **PTZ-C8** | **PTZ-C10** | |
| --- | --- | --- | --- | --- | --- |
| Temperature *T* [K] | 237 | | 225 | 221 | |
| Dielectric strenght *Δε* | 3.67 | | 2.95 | 2.62 | |
| Permittivity in the high-frequency limit *ε_∞_* | 2.43 | | 2.33 | 2.28 | |
| Static permittivity *ε_s_* | 6.10 | | 5.28 | 4.89 | |
| The absolute permittivity of vacuum *ε_0_* [F/m] | 8.85·10^-12^ | | | | |
| Boltzmann’s constant *k_B_* [J/K] | 1.38·10^-23^ | | | | |
| The dipole moment of the isolated molecule *μ* [C·m] | 7.09·10^-30^ | | 7.26·10^-30^ | 7.32·10^-30^ | |
| Assumed density *ρ* [kg/m^3^] | 1176.67 | 1118.75 | | | 1097.34 |
| Molecular weight *M* [kg/mol] | 0.255 | | 0.311 | 0.339 | |
| Avogadro’s numer *N_A_* [1/mol] | 6.02·10^23^ | | | | |
| **Kirkwood correlation factor *g_K_***  *g_K_* = (3·*Δε·ε_0_*·*k_B_*·*T*·*M*)/(*F·μ^2^·ρ·N_A_*)  where *F* = [*ε_s_* (*ε_∞_*+2)^2^]/[3(2 *ε_s_*+ *ε_∞_*)] | **0.84** | | **0.83** | **0.82** | |

1. **Differential scanning calorimetry studies (DSC)**

Calorimetric measurements were carried out using Mettler-Toledo DSC 1 STARe system equipped with a HSS8 ceramic sensor (heat flux sensor with 120 thermocouples) and a liquid nitrogen cooling accessory. The devices were calibrated for temperature and enthalpy using indium and zinc standards. The samples were measured in aluminum crucibles with a 40 μL volume. All DSC experiments were performed with a heating rate equal to 10 K/min over the temperature range from 150 up to 375 K. The glass transition temperatures were determined as the midpoint of the heat capacity increment. The PTZ derivatives tested herein are liquids at room temperature. During the first run, the liquid was rapidly cooled to 150 K (cooling rate 50 K/min). Then, the material was heated (with a heating rate of 10 K/min) up to 375 K. The registered DSC scans are depicted in Figure S1. A characteristic endothermic process corresponding to the glass transition was observed for all samples at T_g_ = 228.68 K, 213.29 K, 209.17 K for PTZ-C4, PTZ-C8, and PTZ-10. The melting and crystallization events were not registered for any substances.

Figure S1. DSC heating scans of PTZ derivatives.

**2. Density measurements**

The density, *ρ*, of studied materials was measured using DMA 4500 M vibrating cell density meter from Anton Paar (Austria). Following the manufacturer’s recommendations, extended calibration of this apparatus was done before measurements with dry air and redistilled water. Importantly, viscosity-related errors were automatically corrected in full range, which was verified using the oil N100 at two temperatures, *i.e.*, 293.15 and 323.15 K. Density was measured in the temperature range from 283.15 to 363.15 K with a step of 5 K, and at least two independent values were obtained at each temperature. Standard uncertainties, *u*, of density, temperature, and pressure are *u*(*T*) = 0.01 K, *u*(*ρ*) = 0.002·*ρ* and *u*(*p*) = 2 kPa, respectively. The uncertainty of density and stated conditions was obtained by the following advice reported in the literature (R.D. Chirico, M. Frenkel, J.W. Magee, V. Diky, C.D. Muzny, A.F. Kazakov, et al. Improvement of quality in the publication of experimental thermophysical property data: challenges, assessment tools, global implementation, and online support, J. Chem. Eng. Data 58 (2013) 2699–2716.) Registered data are presented in Figure S2. The densities were estimated from a linear fit of the measured data.

Figure S2. The temperature dependence of the density for PTZ-C4, PTZ-C8, and PTZ-C10.
